# Does Education Influence Life-Course Depression in Middle-Aged and Elderly in China? Evidence from the China Health and Retirement Longitudinal Study (CHARLS)

**DOI:** 10.3390/ijerph20021256

**Published:** 2023-01-10

**Authors:** Xiwu Xu, Yaodong Zhou, Dai Su, Yuan Dang, Xianwen Zhang

**Affiliations:** 1 School of Economics and Management, Beijing Jiaotong University, Beijing 100091, China; 2 Department of Health Management and Policy, School of Public Health, Capital Medical University, Beijing 100069, China; 3 Department of Global Health, School of Public Health, Peking University, Beijing 100191, China; 4 Beijing Obstetrics and Gynecology Hospital, Capital Medical University Beijing Maternal and Child Health Care Hospital, Beijing 100026, China

**Keywords:** education, depression, life course, middle-aged and elderly, China

## Abstract

Mental health problems have become a major public health problem worldwide and are more common among middle-aged and elderly people in China. Research on the effect of education on depression is limited, and whether the relationship between education and depression changes over the life course remains unclear. This study was based on the cross-sectional data of 15,767 middle-aged and elderly individuals in the 2018 tracking survey (Wave 4) of the China Health and Retirement Longitudinal Study (CHARLS) database. Multiple linear regression and bootstrap methods were developed to detect the mediating effect of education on depression. In all samples or different age groups, education was significantly positively associated with depression. Three mediators (economic level, health-related lifestyle, and cognitive level) were significantly positively associated with depression, and cognitive level had a greater effect on depression than economic level and health-related lifestyle. The total, direct, and indirect effects of the whole samples and elderly samples were significant; however, the direct effect of the middle-aged samples was insignificant, and the total and indirect effects of the three mediating pathways were all significant, that is, economic level, health-related lifestyle, and cognitive level should produce complete mediation. The multiple linear regression and bootstrap methods could successfully detect the mediating effect of education on depression. On the basis of the education, economic level, health-related lifestyle, cognitive level, and depression of middle-aged and elderly people, we established and compared the total, direct, and mediating effects of education on depression under the life course. The mediating variables should be further increased, and the measurement methods of depression should be developed to improve the credibility of the research results.

## 1. Introduction

Mental health problems have become a major public health problem worldwide, and according to the World Health Organisation (WHO) [1], the bulk of the global health burden can be attributed to non-psychiatric illnesses and common mental disorders, such as depression and anxiety. Depression is more common among middle-aged and elderly people in China, with 4.1% and 3.8% of people aged 50–64 and 65 years old, respectively, which are higher than 3.6% and 3.1% of people aged 18–34 and 35–49, respectively. Some studies have found that depression is closely related to many adverse outcomes, and it also brings a heavy medical burden to families and society [2,3]. According to Fan et al. [4], mental illnesses, such as depression, account for more than 20% of the total medical expenses in China. Given the age-related characteristics of depression prevalence and disease manifestations, a series of threats to mental health may expand under the rapidly ageing population in China.

The financial rewards of education have been widely demonstrated by a large number of researchers, and research on the effect of education on health is limited [5]. More recently, several studies have found that education also plays an important role in improving people’s physical health [6,7]. However, as an important dimension for measuring health, mental health has not been fully explored. Some studies have shown higher rates of psychological distress among people with low levels of education, confirming the important relationship between education and depression [8,9]. Some studies have also reported that economic status, lifestyle, and self-efficacy are important mediators between education and depression [10,11]. However, few studies have explored the role of the above mediating variables, and the current research conclusions have not reached a consensus. In addition, whether the relationship between education and depression changes over the life course remains unclear. Some research results only exist at the theoretical level, and the relevant empirical conclusions are inconsistent [12,13]. However, there exist significant differences in the influence of education on health at different ages, and further research is needed on the influence of education on depression in the life course.

Several studies have analysed the association between depression and its factors, which mainly include the following types of indicators: (1) demographic characteristics, such as sex [14], age [15], marital status [16], education [17], place of residence [18], ethnicity [19], and religious belief [20]; (2) economic level, such as personal income [21] and household income [22]; (3) health-related lifestyle, including smoking [23], alcohol consumption [24], social activities [25], physical exercise [26], and taking a nap after lunch [27]; and (4) cognition level, including episodic memory [28] and mental status [29]. However, most studies have only analysed the influence of education on depression from one or several of the above factors, and more comprehensive comparative studies for all factors are lacking [17,18,19,20,21,22,23,24,25,26,27,30,31,32]. Moreover, systematic research on the analysis of the mediating effect of education on depression is lacking, and the available approach only analyses the proportion of each influencing channel and channel in the process of education affecting depression and does not establish a mediation effect model for each mediating variable.

To the best of our knowledge, no previous study has used a mediation effect model combined with longitudinal data to analyse the influence of education on depression and its mediating mechanism from the perspective of life course. Therefore, this study utilises survey data from the China Health and Retirement Longitudinal Study (CHARLS) as a sample to analyse the influence of education on depression and its mediating mechanism from the perspective of life course by using a mediation effect model.

## 2. Materials and Methods

### 2.1. Study Design and Setting

#### 2.1.1. Data Resource

The data used in this study are derived from the 2018 tracking survey (Wave 4) of CHARLS. The CHARLS survey collects a set of high-quality microdata representing families and individuals aged 45 years and older in China to analyse the ageing Chinese population and promote interdisciplinary research on ageing. The CHARLS questionnaire includes demographic backgrounds, family structure and financial support, health status and functioning, healthcare and insurance, work, retirement and pension, income, expenditures and assets, and housing characteristics. The questionnaire design draws on international experience, including the Health and Retirement Study; the English Longitudinal Study of Ageing; and the Survey of Health, Ageing and Retirement in Europe. The project adopted multistage sampling, and probability proportionate to size sampling methods were adopted in the county/district and village sampling stages. CHARLS pioneered the electronic drawing software (CHARLS-GIS) technology and used the map method to create village-level sampling frames. The CHARLS baseline survey was launched in 2011 and covered 150 county-level units, 450 village-level units, and 17,708 individuals from 10,257 households in 28 provinces, municipalities, and autonomous regions (Table 1).

Generally, the CHARLS baseline is a good representation of the elderly population of China. Sampling is conducted every 2–3 years, the latest tracking survey of CHARLS was conducted and published in 2018, and the fifth of survey data in 2021 has not yet been released. Counting the refresher samples and age-eligible respondents who failed to be found in the baseline but were successfully contacted in the follow-up waves shows that the total number of individuals (main respondents plus spouses) has increased from 17,708 in Wave 1 to 19,817 in Wave 4.

**Figure 1:**
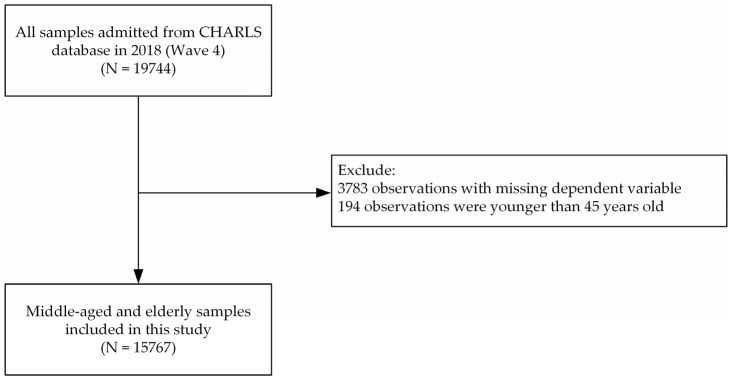
Selection of study participants. The N stands for the observations.

In this study, 19,744 observations were admitted from the CHARLS database in Wave 4. After selection, the remaining 15,767 observations were used in this paper. The specific sample selection process is shown in Figure 1.

#### 2.1.2. Theoretical Framework

**Figure 2:**
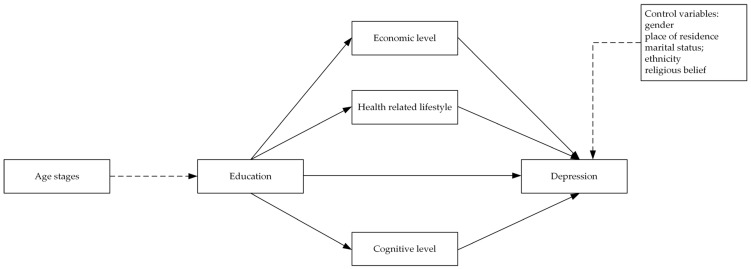
A theoretical framework for the impact of education on depression.

On the basis of some research results, this study proposed a theoretical framework. We believe that education has a significant effect on depression, and this influence is mainly transmitted through three types of factors represented by economic level, lifestyle, and cognitive level. This relationship and its mediating mechanism may vary in different age stages. Figure 2 shows the architecture of our theoretical framework.

Therefore, this study assessed the association between education and life-course depression in middle-aged and elderly in China. We propose four hypotheses: (1) education can indirectly affect depression in middle-aged and elderly people through economic level, (2) education can indirectly affect depression in middle-aged and elderly people through health-related lifestyle, (3) education can indirectly affect depression in middle-aged and elderly people through cognitive level, and (4) age stage in the life course moderates the effect of education on depression.

### 2.2. Study Variables

#### 2.2.1. Outcome Variable

Depression was measured by the Centre for Epidemiological Studies Depression Scale-10 (CES-D10) in the CHARLS questionnaire [26]. The CES-D10 comprises 10 questions about depression, and the answers included four options: (1) rarely, (2) some days (1–2 days per week), (3) occasionally (3–4 days per week), and (4) most of the time (5–7 days per week). Among the 10 questions, 8 questions were negative statements, and 2 were positive statements. The answers were recorded as 0 (rarely) to 3 (most of the time) for the negative questions and 3 (rarely) to 0 (most of the time) for the positive questions. The depression index was obtained from the sum of the scores of the 10 questions. We calculated the total score of 10 items as a respondent’s final score, with a maximum of 30 points. A higher score indicated lower depression.

#### 2.2.2. Mediators

This study selected 8 variables of the respondents in the 2018 tracking survey as mediators of depression, including the following: (1) economic level, such as personal annual gross income (a continuous variable, taking a logarithm); (2) health-related lifestyle, including smoking (yes vs. no), alcohol consumption (yes vs. no), social activities (yes vs. no), physical exercise (yes vs. no), and taking a nap after lunch (yes vs. no); and (3) cognition level, including episodic memory (a continuous variable) and mental status (a continuous variable).

Many studies have divided cognitive ability into two aspects: variable and fixed abilities, which can be represented by episodic memory ability and mental status, respectively [33,34]. The interviewer would read 10 words to the respondents and ask the respondents to recall the words they heard at two different times. If they successfully recalled a word, then they would get 1 point; otherwise, they would get 0 points. The interviewer asked the respondents twice, and the average of the two recall scores was the episodic memory score. Therefore, the episodic memory score ranged from 0 to 10 points. Mental status was measured by the number of five math calculation questions answered correctly by the respondents and whether they knew the year, month, day, week, and season of the interview. Each correct answer was scored 1 point, and a wrong answer was scored 0 points. The total mental state score ranged from 0 to 5. Therefore, cognitive ability was measured using the sum of episodic memory and mental state scores, which ranged from 0 to 15.

#### 2.2.3. Control Variables

The control variables in this study mainly consisted of demographic characteristics, such as gender (male vs. female), age groups (a categorical variable), place of residence (city/town vs. rural), marital status (married with spouse presently vs. married but not living with spouse temporarily for reasons such as work/separated/divorced/widowed/never married), ethnicity (Han vs. ethnic minorities), and religious belief (yes vs. no).

#### 2.2.4. Key Variables

The key variables included education, which was measured by the highest level of education, and age, which was divided into middle and old age according to the age classification criteria issued by the United Nations Health Organisation. In this study, the samples aged 45–59 were divided into the middle-aged group, and the samples aged 60 and above were divided into the middle-aged and elderly groups.

The participants were asked, “What is the highest level of education you have completed?” The original answers were classified into 11 categories: illiterate, did not finish primary school but capable of reading and/or writing, home school, elementary school, middle school, high school, vocational school, three-year college, four-year college, master’s programs, and Ph.D. programs. A higher number indicated a higher level of education.

The descriptive statistics of each variable are shown in Table 2.

### 2.3. Data Analysis

#### 2.3.1. Processing of Missing Values

We used the k-nearest neighbour (k-NN) imputation algorithm to fill in the missing data in our study (Table 1), where each missing value on some records was replaced by a value obtained from related cases in the entire set of records. The most notable features of the k-NN imputation algorithm are as follows: (a) the imputed value is the value that has actually appeared, and no secondary processing is performed; (b) the distribution structure of the original data is retained in accordance with the variable information; and (c) k-NN imputation is completely nonparametric and does not depend on the relationship between y and x. We assumed that the k-NN method determines the closest k (k = 5) elderly with missing data in accordance with the Euclidean or L2 distance.

#### 2.3.2. Test of Mediating Effect

According to the theoretical framework, this study established a multiple linear regression equation of the influence of education on depression to detect the mediating effect, as shown as follows:

H_i_ = α_0_ + α_1_E_i_ + X_i_θ + ε_i_ (1)

where H_i_ represents the depression of individual i, E_i_ is the education of individual i, X_i_ represents all control variables of individual i, and ε_i_ represents the random disturbance term.

H_i_ = β_0_ + β_1_E_i_ + X_i_θ + M_i_γ + μ_i_ (2)

In Equation (2), this study gradually increased the mediating variable M. M_i_ represents the value of individual i on the three mediators, and μ_i_ represents the random disturbance term. The three mediators in this study are economic level, health-related lifestyle, and cognitive level.

H_i_ = δ_0_ + δ_1_E_i_ + δ_2_EA_i_ + X_i_θ + M_i_γ + ρ_i_ (3)

In Equation (3), to reflect the influence of education on depression in the life course, we further included the interaction item EA between education and different age stages. EA_i_ represents the interaction term results of different education levels and age stages of individual i, and ρ_i_ represents the random disturbance term.

Given that this study involves multiple parallel mediators and the distribution of the health level of the dependent variable is biased, we further used the bootstrap method based on ordinary least square regression analysis to conduct an accurate mediation test for our hypothesis. The number of bootstrap repeated samplings set in this paper was 1000 times.

Stata 14.0 software (Stata Corp LP, College Station, TX, USA) was used for statistical analysis in a Windows environment.

## 3. Results

### 3.1. Influence of Education on the Depression of All Samples Using Linear Regression

Table 3 shows the effect of education on depression based on all samples. In Model 1, education was significantly positively associated with depression (α_1_ = 0.520, *p*-value < 0.001). Model 2 added the economic level variable based on Model 1. The degree of the significant positive association between education and depression decreased (β_11_ = 0.455, *p*-value < 0.001). This situation also appears in Models 3 and 4, which respectively added health-related lifestyle (β_12_ = 0.402, *p*-value < 0.001) and cognitive level (β_13_ = 0.505, *p*-value < 0.001). The explanation level of the three mediators for depression could reach 78.5%, and they were all significantly positively associated with depression. The cognitive level had a greater influence on depression than economic level and health-related lifestyle (0.658 vs. 0.125/0.058).

Model 6 further added the interaction term of education and age group based on Model 5. Education was significantly positively associated with the depression of middle-aged and elderly people (β_15_ = 0.152, *p*-value < 0.05), but no significant difference was observed between the two age groups (δ_1_ = −0.024, *p*-value = 0.449).

Table 4 shows the results of the correlation test of the core variables in this study. It can be seen that, except for the significant negative correlation between health-related lifestyle and education, and the interaction between age and education, all other variables are positively correlated.

### 3.2. Influence of Education on the Depression of the Middle-Aged and Elderly Using Linear Regression

As shown in Table 5, this study analysed middle-aged and elderly samples. The results showed that the effect of education on depression had a significantly positive influence in both groups, indicating that with the change of age groups, the effect of education on depression was not significant. Education had a significant positive effect on depression through three mediators in all age groups. Consistent with the full sample results, the cognitive level was more important than the other two mediators.

### 3.3. Results of Multiple Mediation Tests: Total, Direct and Indirect Effect

**Figure 3:**
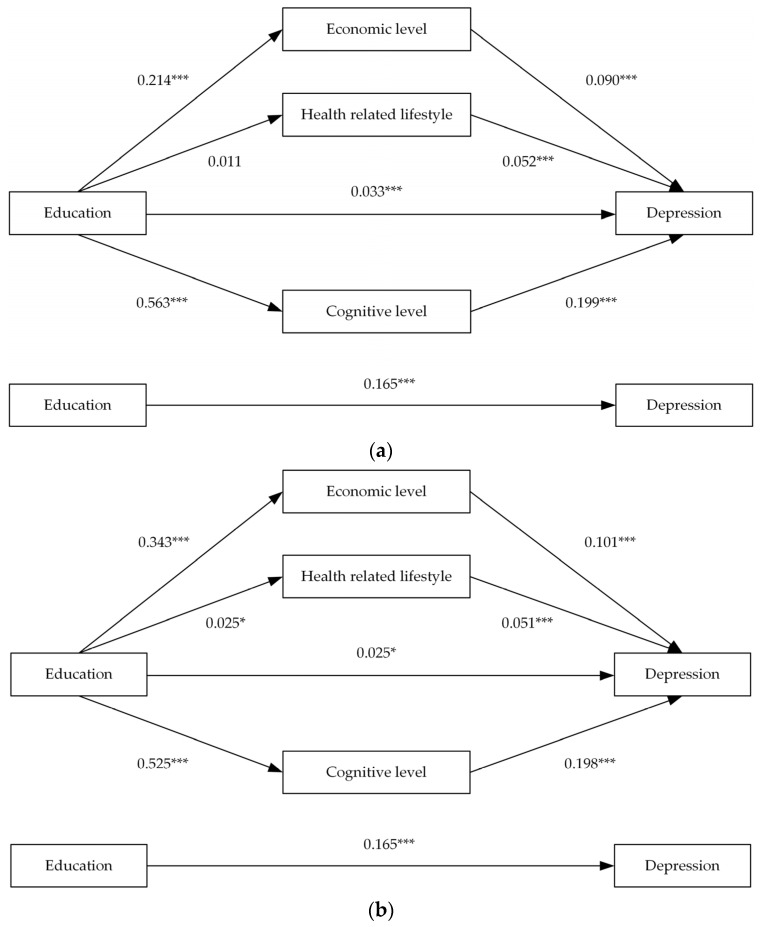

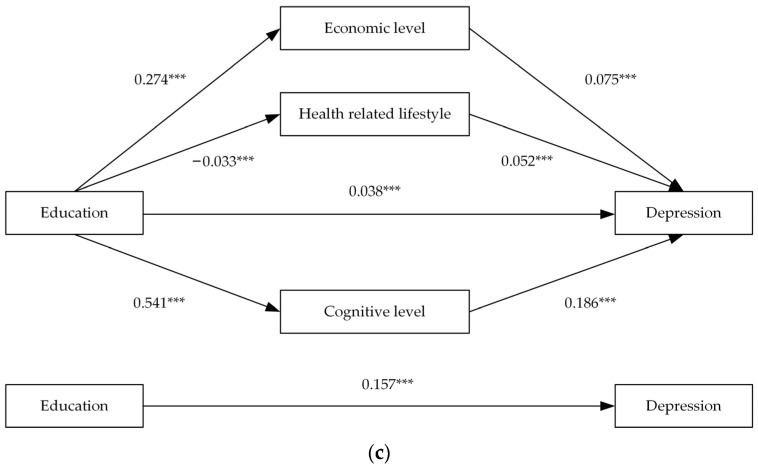
Results of multiple mediation analysis. (**a**) All samples; (**b**) Middle-aged samples; (**c**) Elderly samples. *** represents *p*-value < 0.01, * represents *p*-value < 0.1.

This study used the bootstrap method to further analyse the multiple mediating outcomes of education on depression. From Table 6 and Figure 3a, the total, direct and indirect effects of the whole sample were significant, and the cognitive level path accounted for 84.9% of the indirect effect. From Table 6 and Figure 3b, the direct effect of the middle-aged sample was not significant, and the total and indirect effects of the three mediating pathways were all significant; that is, economic level, health-related lifestyle, and cognitive level should produce complete mediation. The cognitive level path accounted for 74.3% of the indirect effect. As shown in Table 6 and Figure 3c, similar to the whole sample, the total, direct, and indirect effects of the elderly were all significant, indicating that in the elderly sample, the three mediating pathways only produced a partial mediating effect. The cognitive level path accounted for 83.9% of the total indirect effect.

## 4. Discussion

This study was based on cross-sectional data from 15,767 middle-aged and elderly individuals in the 2018 follow-up survey of the CHARLS database. The data included the sociodemographic information, economic level, cognitive level, health-related lifestyle, and depression of each middle-aged and elderly sample. We analysed the effect of education on depression using multiple linear regression models and bootstrap multiple mediation models. The results showed that the two methods showed good performance in analysing the relationship between education and depression and the mediating effect. There existed a mediating effect in the influence of education on depression, and significant differences were observed in the mediating effect in the life course.

This study found that three mediating variables, namely, economic level, health-related lifestyle, and cognitive level, had significant positive effects on depression, which is consistent with the findings of multiple studies [35,36]. Previous studies on the relationship between education and health have not analysed depression separately and rarely considered the mediating effect of cognitive level [37,38,39]. The concept of life course in previous studies has mainly focused on the comparison between young and older samples [40,41]. This study further refined the research samples, mainly comparing middle-aged and elderly groups, and enriched the life course perspective of related research.

This study found that economic level, health-related lifestyle, and cognitive level were all important mediating variables affecting the depression of middle-aged and elderly people. The explanation rate could reach about 80%, and cognitive level was the most important. The effects of the three mediating variables were different between the middle-aged and elderly groups. The influence of the economic level in the middle-aged group was significantly lower than that of the health-related lifestyle, but in the elderly group, the influence of the two mediating variables was basically the same. Thus, the economic level is more likely to appear in the treatment after the disease occurs, whereas the health-related lifestyle focuses on the prevention of the disease. In addition, the cognitive level was the most important in the middle-aged and elderly groups, which indicates that if there exists a problem with the cognitive level in the life course, it will have a devastating effect on mental health and quality of life [42]. Each person has unique core values and priorities. The degree of depression is determined by how well people achieve core values in their lives [43,44].

The results of the mediation effect model analysis showed that the direct effect of education on the depression of middle-aged people was insignificant, but it was significant for the elderly sample; the former was a complete mediator, whereas the latter was a partial mediation [45]. To a certain extent, this finding reflects that the ways in which education affects depression will gradually diversify with the development of the life course. In addition to health-related lifestyle, economic level, and cognitive level, education also affected the depression of the elderly sample through other factors, which may include dietary nutrition, sleep quality, social support, and medical and pension insurance [46]. In addition, no significant difference was observed in the influence of education on the depression of middle-aged and elderly people under the life course. However, some studies have shown that the effect of education on depression differs significantly between young and older people, which suggests that the cumulative advantage of education on depression is mainly reflected at about 45 years of age, and the advantage then gradually weakens; the reason is the main knowledge reserve is continuously strengthened in the youth, which has the greatest influence on depression [47]. Thereafter, the knowledge reserve in the middle-aged and elderly is relatively stable, so the influence on depression will not change significantly.

This work is the first comprehensive study of the relationship between depression and education in middle-aged and elderly people across the life course using national-level data. The results of this study based on the mediation model can help policymakers and the general public provide a scientific basis for early intervention in education.

## 5. Limitations

Our study has some limitations. Firstly, only three mediators and six control variables were considered. We limited our analysis to potential mediator variables already available in the dataset, including economic level, health-related lifestyle, and cognitive level. Therefore, the generalisability of our conclusions still needs to be further strengthened, such as cultural factors. In future studies, we could continue to add more specific mediators to help improve model performance, such as educational equality, dietary nutrition, and sleep quality. Secondly, the cross-sectional data mainly used in this study may lead to biased conclusions, and the panel data could be used to further verify the conclusions in the next step. Finally, the measurement of education indicators in this study was mainly based on school education; however, the learning and accumulation of life experience and health experience are also highly important. Due to data limitations, enriching the connotation of education indicators is impossible. In the next step, we could collect more variables to comprehensively evaluate the education.

## 6. Conclusions

On the basis of the education, economic level, health-related lifestyle, cognitive level, and depression of middle-aged and elderly people, we established and compared the total, direct, and mediating effects of education on depression under the life course. Mediating variables (economic level, health-related lifestyle, cognitive level) have significant effects on depression, and the strength and criticality of education and the three mediator variables in the life course are also different. The number of mediating variables should be further increased, and the measurement methods of depression must be developed to improve the credibility of research results.

## Figures and Tables

**Table 1:** The 28 provinces from which the CHARLS baseline survey samples came from.

Province (1–14)	Province (15–28)
Anhui	Jiangsu
Beijing	Jiangxi
Chongqing	Jilin
Fujian	Liaoning
Gansu	Qinghai
Guangdong	Shandong
Guangxi	Shanghai
Guizhou	Shannxi
Henan	Shanxi
Hebei	Sichuan
Heilongjiang	Tianjin
Hunan	Xinjiang
Hubei	Yunnan
Inner mongolia	Zhejiang

**Table 2:** Demographic characteristics of samples in this study.

Variables	Mean	Std. Dev.	Min	Max
Age	60.89	9.30	45	108
Gender	0.49	0.50	0	1
Place of residence	0.29	0.45	0	1
Marital status	0.81	0.39	0	1
Ethnicity	0.92	0.27	0	1
Religious belief	0.10	0.30	0	1
Education	2.62	1.91	0	10
Depression	21.57	6.49	0	30
Personal annual gross income (logarithm)	2.66	1.84	0	6.78
Smoking	0.43	0.50	0	1
Alcohol consumption	0.36	0.48	0	1
Social activities	0.56	0.50	0	1
Physical exercise	0.92	0.27	0	1
Taking a nap after lunch	0.62	0.49	0	1
Episodic memory	4.23	1.85	0	10
Mental status	3.83	1.25	0	5

**Table 3:** Results of multiple regression analysis and mediation effect analysis of all samples.

Variables	Model 1 Basic Variables	Model 2 + Economic Level	Model 3 + Health Related Lifestyle	Model 4 + Cognitive Level	Model 5 All Variables	Model 6 + Cross Terms
Education	0.520 *** (0.030)	0.455 *** (0.030)	0.490 *** (0.030)	0.178 *** (0.033)	0.112 *** (0.034)	0.152 ** (0.056)
Economic level		0.349 *** (0.030)			0.312 *** (0.030)	0.305 *** (0.029)
Health related lifestyle			0.402 *** (0.054)		0.313 *** (0.054)	0.313 *** (0.054)
Cognitive level				0.505 *** (0.023)	0.481 *** (0.023)	0.483 *** (0.023)
Education Age						−0.024 (0.032)
Explanation (1 − β_1_/α_1_)		0.125	0.058	0.658	0.785	
Control variables	Yes	Yes	Yes	Yes	Yes	Yes
Constant	17.875 *** (0.275)	17.973 *** (0.274)	16.451 *** (0.336)	14.455 *** (0.313)	13.598 *** (0.359)	13.414 *** (0.307)
R^2^/pseudo R^2^	0.080	0.087	0.083	0.107	0.115	0.115
Number of observations	15,767	15,767	15,767	15,767	15,767	15,767

Note: *** represents *p*-value < 0.001, ** represents *p*-value < 0.05.

**Table 4:** Correlation tests for key variables.

Variables	Education	Economic Level	Health Related Lifestyle	Cognitive Level	Education Age
Education	1.000				
Economic level	0.274 ***	1.000			
Health related lifestyle	−0.033 **	0.067	1.000		
Cognitive level	0.541 ***	0.234 ***	0.067 ***	1.000	
Education Age	0.990 ***	0.272 ***	−0.033 **	0.548 ***	1.000

Note: *** represents *p*-value < 0.001, ** represents *p*-value < 0.05.

**Table 5:** Results of multiple regression analysis and mediation effect analysis of middle-aged samples and elderly samples, respectively.

Variables	The Middle-Aged Samples				The Elderly Samples			
	Model 1 Basic Variables	Model 2 + Economic Level	Model 3 + Health Related Lifestyle	Model 4 + Cognitive Level	Model 1 Basic Variables	Model 2 + Economic Level	Model 3 + Health Related Lifestyle	Model 4 + Cognitive Level
Education	0.550 *** (0.043)	0.453 *** (0.044)	0.518 *** (0.043)	0.183 *** (0.048)	0.496 *** (0.042)	0.450 *** (0.042)	0.470 *** (0.042)	0.173 *** (0.047)
Economic level		0.328 *** (0.035)				0.430 *** (0.057)		
Health related lifestyle			0.357 *** (0.078)				0.432 *** (0.075)	
Cognitive level				0.537 *** (0.034)				0.486 *** (0.032)
Education Age								
Explanation (1 − β1/α1)		0.176	0.058	0.667		0.093	0.052	0.651
Control variables	Yes	Yes	Yes	Yes	Yes	Yes	Yes	Yes
Constant	17.637 *** (0.313)	17.423 *** (0.312)	16.380 *** (0.417)	14.275 *** (0.374)	17.450 *** (0.313)	16.407 *** (0.342)	15.963 *** (0.406)	15.171 *** (0.343)
R^2^/pseudo R^2^	0.082	0.093	0.085	0.113	0.076	0.082	0.079	0.101
Number of observations	7424	7424	7424	7424	8343	8343	8343	8343

Note: *** represents *p*-value < 0.01.

**Table 6:** Results of multiple mediation tests: total, direct, and indirect effects.

Samples	All Type of Effect	Estimate	Bias-Corrected Percentile (95%CI)		*p*-Value	Number of Observations
			Lower	Upper		
All	Total effect	0.165	0.147	0.181	0.003	15,767
	Direct effect	0.033	0.014	0.053	0.003	15,767
	Indirect effect	0.132	0.121	0.143	0.002	15,767
	education→economic level	0.214	0.200	0.229	0.001	15,767
	education→health related lifestyle	0.011	−0.004	0.025	0.166	15,767
	education→cognitive level	0.563	0.553	0.573	0.002	15,767
	economic level→depression	0.090	0.073	0.105	0.002	15,767
	health related lifestyle→depression	0.052	0.035	0.070	0.002	15,767
	cognitive level→depression	0.199	0.181	0.217	0.002	15,767
middle-aged	Total effect	0.165	0.141	0.190	0.002	7424
	Direct effect	0.025	−0.003	0.054	0.084	7424
	Indirect effect	0.140	0.125	0.155	0.002	7424
	education→economic level	0.343	0.324	0.363	0.002	7424
	education→health related lifestyle	0.025	0.004	0.047	0.027	7424
	education→cognitive level	0.525	0.508	0.541	0.002	7424
	economic level→depression	0.101	0.077	0.123	0.003	7424
	health related lifestyle→depression	0.051	0.026	0.076	0.002	7424
	cognitive level→depression	0.198	0.174	0.225	0.001	7424
elderly	Total effect	0.157	0.132	0.180	0.003	8343
	Direct effect	0.038	0.012	0.063	0.005	8343
	Indirect effect	0.120	0.104	0.136	0.002	8343
	education→economic level	0.274	0.253	0.294	0.002	8343
	education→health related lifestyle	−0.033	−0.053	−0.013	0.002	8343
	education→cognitive level	0.541	0.527	0.554	0.002	8343
	economic level→depression	0.075	0.052	0.079	0.002	8343
	health related lifestyle→depression	0.052	0.029	0.076	0.002	8343
	cognitive level→depression	0.186	0.160	0.212	0.002	8343

## Data Availability

The original data of the CHARLS database is open to the public and can be obtained through an application. The official website of CHARLS is http://charls.pku.edu.cn/, accessed on 5 December 2022. The datasets used and/or analysed during the current study are available from the corresponding author upon reasonable request.
